# Supplementary material for: MQPD: An Antioxidant Quinone–Dopamine Hybrid Which Induces Sustained Brain Dopamine Elevation
Source: Antioxidants (Basel). 2025 Nov 27;14(12):1416. doi: 10.3390/antiox14121416 (PMC12729426; doi:10.3390/antiox14121416)
Supplement: Supplementary file 1 [file antioxidants-14-01416-s001.zip › antioxidants-3947199-supplementary.pdf]

A027.032.{1H}_001000fid

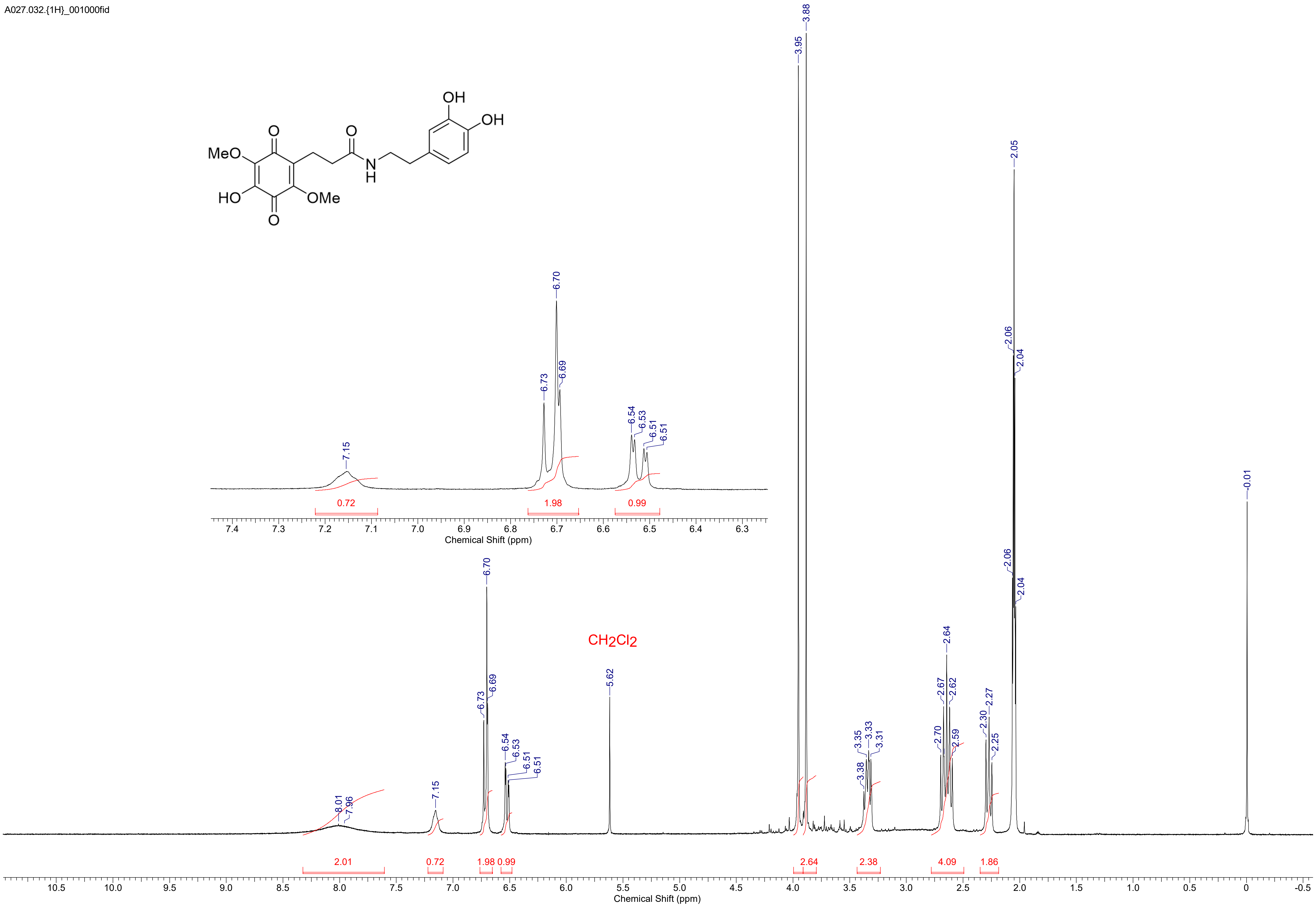

Figure S1: ^{1}H NMR spectrum of MQPD using Acetone-d_6 as the NMR solvent

AO27.033.{13C}_001000fid

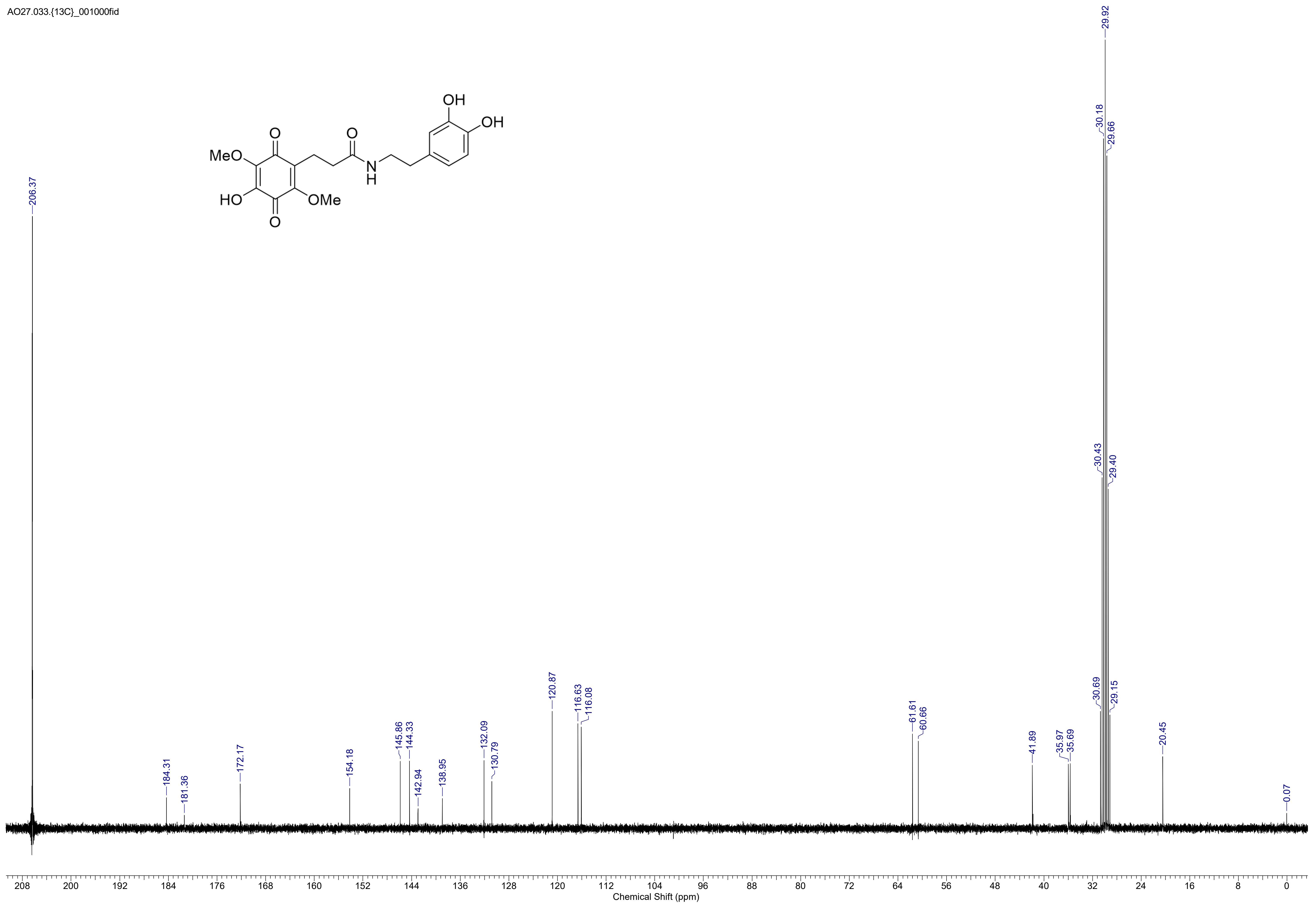

Figure S2: ^{13}C NMR spectrum of MQPD using Acetone-d_6 as the NMR solvent

Display Report

Analysis Info

Analysis Name	D:\Data\Kolotyrkina\2025\Adaeva\0707031.d	Acquisition Date	07.07.2025 16:04:34
Method	tune_low.m	Operator	BDAL@DE
Sample Name	/DEMC 027.032	Instrument / Ser#	micrOTOF 10248
Comment	C19H21NO8 clb added CH3OH		

Acquisition Parameter

Source Type	ESI	Ion Polarity	Positive	Set Nebulizer	0.4 Bar
Focus	Not active			Set Dry Heater	180 °C
Scan Begin	50 m/z	Set Capillary	4500 V	Set Dry Gas	4.0 l/min
Scan End	3000 m/z	Set End Plate Offset	-500 V	Set Divert Valve	Waste

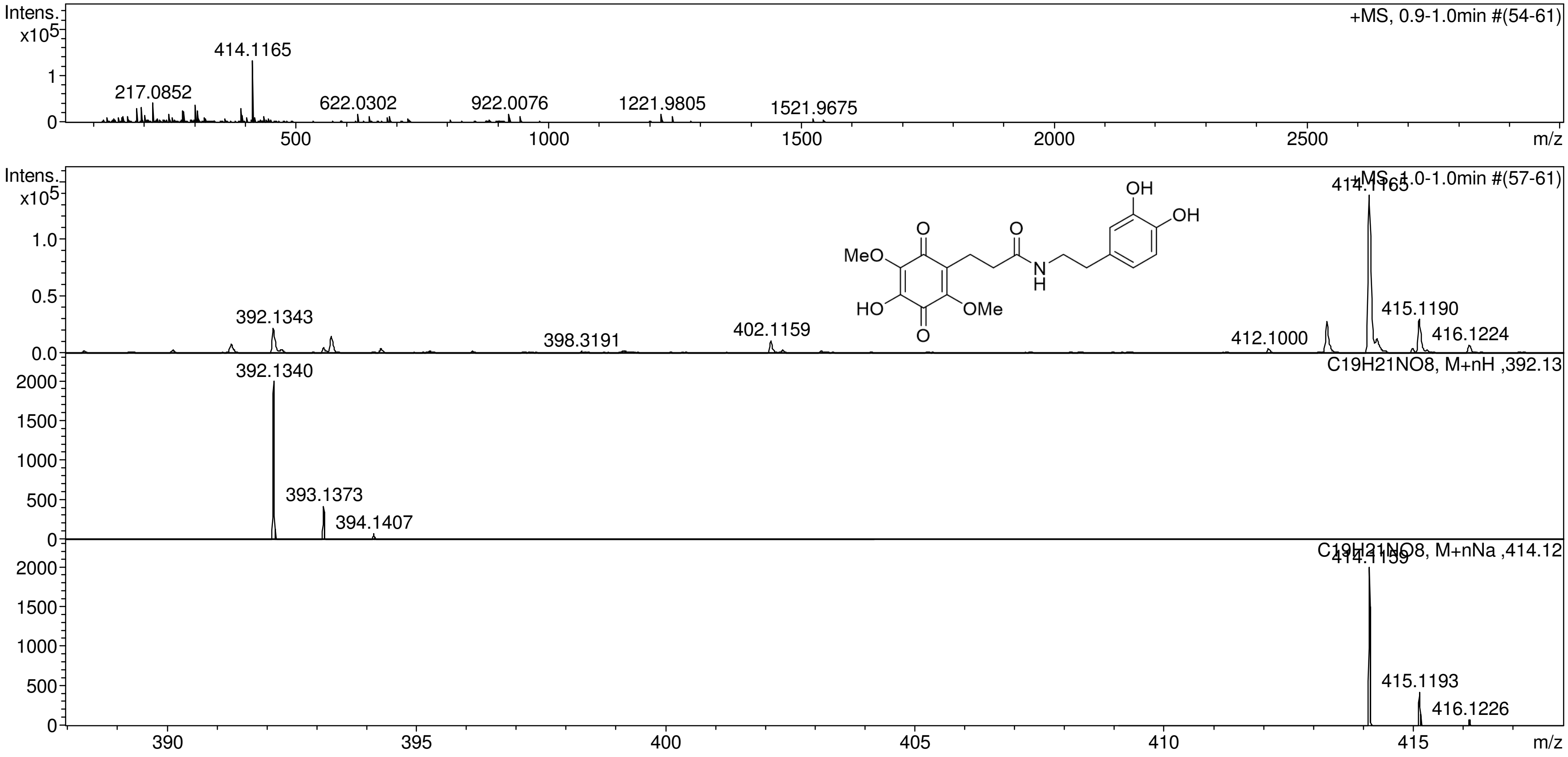

Figure S3: HRMS spectrum of MQPD

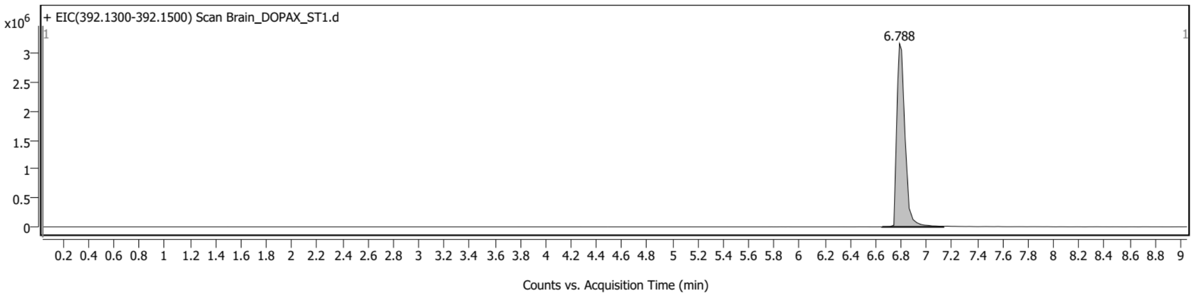

Chromatogram Peaks

Peak	Start	RT	End	Height	Area	Area %
1	6.650	6.788	7.134	3172298	14275313	100.00

Sample Spectra

+ Product Ion (rt: 6.747-6.990 min)
(392.1323 -> **)

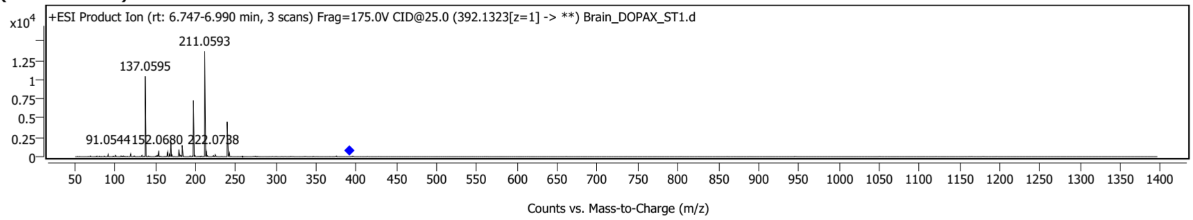

Spectrum Peaks

m/z	Z	Abund	Abund %	m/z (Calc)	Diff (ppm)	Ion Species	Formula	Ion Type
91.0544		298	2.14					
100.1118		163	1.17					
119.0489		357	2.57					
133.0267		164	1.18					
137.0595	1	10455	75.11					
138.0619	1	812	5.83					
152.0680		161	1.16					
154.0856		804	5.78					
165.0534		601	4.32					
167.0698		361	2.60					
169.0488	1	1988	14.28					
170.0513	1	268	1.93					
179.0329	1	951	6.83					
180.0398	1	162	1.16					
180.0637		284	2.04					
182.0438		148	1.06					
183.0641		1487	10.68					
197.0432	1	7672	55.12					
198.0481	1	727	5.23					
199.0576	1	157	1.13					
211.0593	1	13919	100.00					
212.0625	1	1844	13.25					
213.0746	1	710	5.10					
214.0786	1	155	1.12					
222.0738		143	1.03					
224.0292		277	1.99					
239.0538	1	4621	33.20					
240.0570	1	614	4.41					
242.0677		533	3.83					

Figure S4 Typical high-resolution XIC trace for MQPD ([M+H]+ m/z 392.134) and its fragmentation spectrum.

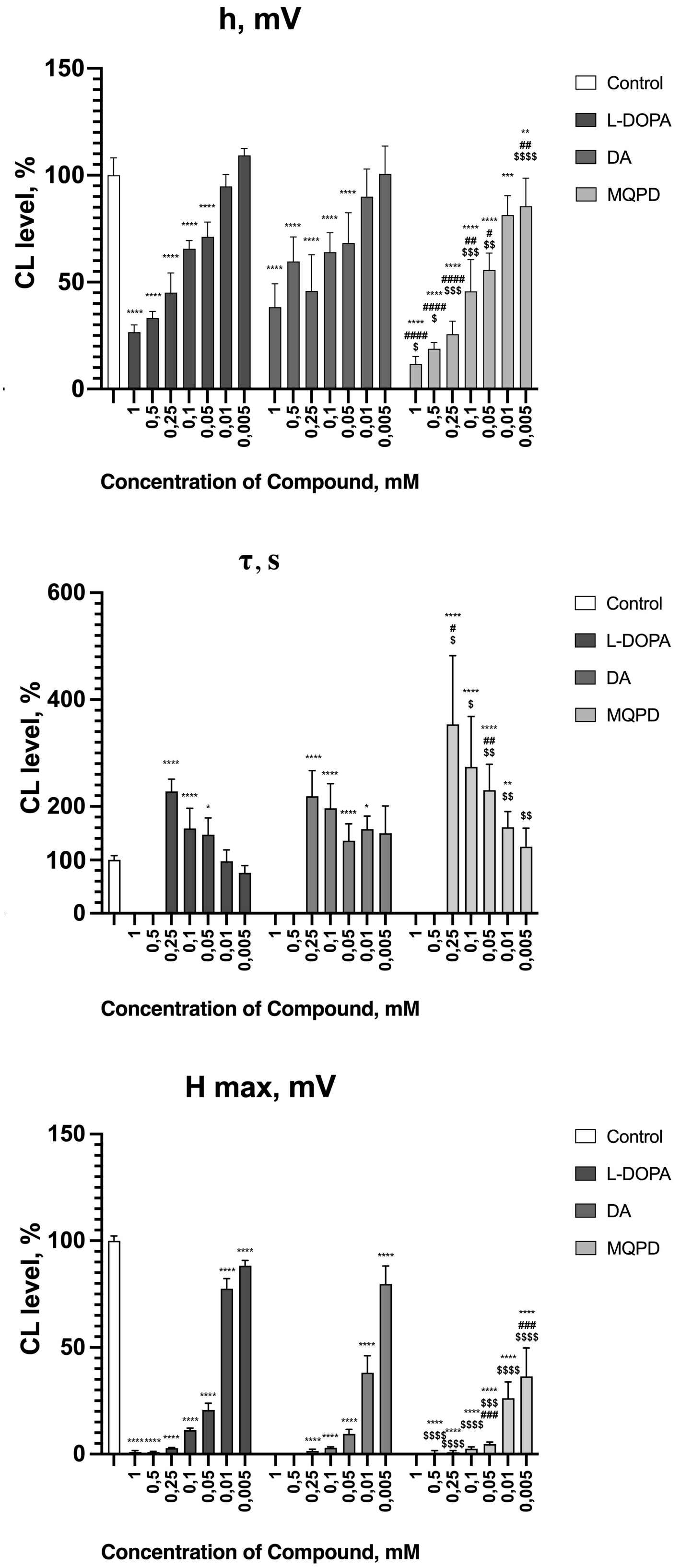

Figure S5. Parameters of iron-induced lipoprotein oxidation. (h) lipid hydroperoxide content, represented by the fast flash amplitude (mV); (τ) antioxidant activity, indicated by the lag period duration (s); and (H) total oxidizability, shown by the maximum chemiluminescence intensity (mV). Data are expressed as a percentage of the mean control value (M ± SD) (n = 5-7 samples per group). Statistical significance is denoted as follows: Statistical significance is denoted as * - $p_{adj} < 0.05$, ** - $p_{adj} < 0.01$, *** - $p_{adj} < 0.001$, and **** - $p_{adj} < 0.0001$; # - significant differences from the dopamine (DA) group; $ - significant differences from the LDOPA group.

Tables S1-S6

MQPD content in blood plasma (fig. 11A)

	0,25	0,5	1	3	6	8	24	48	72	96
Number of values	8	7	9	6	5	5	6	6	6	6
Minimum	10839	3671	622,9	4,750	2,510	0,000	0,000	8,770	0,000	0,000
25% Percentile	12170	4642	756,8	12,58	5,600	0,7850	13,13	10,54	2,633	4,770
Median	17785	5402	1371	23,65	10,14	4,270	28,22	14,87	13,19	8,100
75% Percentile	24450	6927	2315	52,55	19,59	31,43	39,50	16,35	14,84	9,668
Maximum	27561	7749	4476	56,49	24,67	54,78	68,01	17,67	16,70	9,960
Range	16722	4078	3853	51,74	22,16	54,78	68,01	8,900	16,70	9,960
Mean	18322	5722	1703	29,16	12,10	13,74	28,66	13,87	10,13	7,015
Std. Deviation	6332	1451	1215	20,80	8,235	23,15	22,35	3,310	6,708	3,681
Std. Error of Mean	2239	548,4	404,9	8,491	3,683	10,35	9,123	1,351	2,739	1,503

MQPD content in brain (fig. 11B)

	0,25	0,5	1	3	6	8	24	48	72	96
Number of values	8	7	9	6	6	6	6	6	6	6
Minimum	10,09	0,8900	0,000	0,000	0,000	0,000	0,000	0,000	0,000	0,000
25% Percentile	12,75	1,490	0,7750	0,000	0,1350	0,2100	0,000	0,000	0,000	0,000
Median	15,27	4,100	1,600	0,000	0,2650	0,2950	0,1800	0,1250	0,1250	0,05000
75% Percentile	24,95	7,150	2,565	0,5425	0,3700	0,3950	0,4475	0,4225	0,4350	0,2925
Maximum	36,14	18,81	5,690	1,030	0,4600	0,5000	0,6500	0,7600	0,8400	0,3000
Range	26,05	17,92	5,690	1,030	0,4600	0,5000	0,6500	0,7600	0,8400	0,3000
Mean	18,73	5,651	1,906	0,2350	0,2517	0,2883	0,2317	0,2200	0,2317	0,1150
Std. Deviation	8,742	6,195	1,723	0,4181	0,1552	0,1635	0,2737	0,2986	0,3274	0,1447
Std. Error of Mean	3,091	2,342	0,5745	0,1707	0,06337	0,06675	0,1117	0,1219	0,1337	0,05909

Striatal DA content (fig. 12A)

	0	0,25	0,5	1	3	6	8	24	48	72	96
Number of values	6	8	6	9	6	6	6	6	6	6	6
Minimum	392,0	490,9	619,4	498,2	564,9	431,4	796,9	1248	1080	1154	541,4
25% Percentile	506,7	522,9	712,2	516,6	630,4	499,0	939,1	1289	1094	1329	953,7
Median	558,8	695,2	815,6	613,3	731,6	584,3	1160	1369	1384	1507	1212
75% Percentile	593,1	1030	1369	911,8	848,5	646,4	1431	1556	1511	1881	1258
Maximum	633,2	1954	1430	1530	1159	660,5	1618	2006	1668	1989	1322
Range	241,2	1464	810,4	1032	594,5	229,1	821,2	757,7	587,9	834,7	780,9

Mean	544,6	871,5	962,1	754,5	764,1	570,6	1181	1450	1346	1565	1103
Std. Deviation	80,99	486,6	339,7	343,9	205,4	90,34	298,9	279,0	224,3	314,0	284,9
Std. Error of Mean	33,06	172,1	138,7	114,6	83,87	36,88	122,0	113,9	91,56	128,2	116,3

Striatal DOPAC content (fig. 12B)

	0	0,25	0,5	1	3	6	8	24	48	72	96
Number of values	6	8	7	9	6	6	6	6	6	6	6
Minimum	49,37	69,54	66,45	70,10	65,40	86,22	89,93	209,6	198,8	205,4	115,9
25% Percentile	60,29	90,57	67,30	80,94	88,23	88,12	119,4	333,6	208,4	208,6	172,8
Median	88,75	107,3	104,6	101,4	110,6	103,1	147,6	401,0	280,5	295,1	234,9
75% Percentile	109,1	194,7	186,1	148,2	185,6	132,3	186,0	484,4	336,7	307,0	325,2
Maximum	134,3	380,9	299,9	383,4	288,9	159,4	246,4	682,3	342,5	320,6	345,5
Range	84,93	311,4	233,4	313,2	223,5	73,17	156,5	472,7	143,6	115,2	229,6
Mean	87,64	151,5	133,7	134,7	137,1	110,6	154,5	414,6	274,8	271,4	240,2
Std. Deviation	29,63	102,5	83,38	99,29	79,80	27,64	53,13	152,1	60,09	50,35	84,86
Std. Error of Mean	12,10	36,23	31,51	33,10	32,58	11,28	21,69	62,11	24,53	20,55	34,64

Striatal HVA content (fig. 12C)

	0	0,25	0,5	1	3	6	8	24	48	72	96
Number of values	6	8	6	9	6	6	6	6	6	6	6
Minimum	40,83	48,37	60,90	39,10	40,42	32,79	53,00	94,65	71,05	102,6	75,30
25% Percentile	43,51	52,17	65,77	53,21	53,69	44,68	59,95	120,4	90,03	113,3	77,10
Median	47,26	67,94	79,99	62,57	61,41	53,73	95,36	146,1	107,8	130,7	120,2
75% Percentile	60,13	92,82	137,4	103,1	95,75	65,32	110,5	166,0	123,8	145,9	154,5
Maximum	86,14	133,8	139,9	204,2	131,0	71,29	141,2	199,5	134,2	160,9	199,3
Range	45,31	85,43	78,97	165,1	90,56	38,50	88,20	104,8	63,15	58,27	124,0
Mean	52,89	75,23	94,12	83,73	72,72	53,92	91,23	145,0	106,2	130,5	122,1
Std. Deviation	16,67	28,93	35,51	51,06	31,76	13,50	31,44	34,32	21,55	19,96	46,14
Std. Error of Mean	6,806	10,23	14,50	17,02	12,97	5,510	12,84	14,01	8,797	8,150	18,83

Striatal 3-MT content (fig. 12D)

	0	0,25	0,5	1	3	6	8	24	48	72	96
Number of values	6	8	7	9	6	6	6	6	6	6	6
Minimum	27,23	29,18	24,41	15,04	23,65	25,57	35,30	64,34	57,40	63,01	36,64
25% Percentile	29,37	30,99	34,49	27,01	31,19	27,10	41,85	64,96	57,58	68,09	40,30
Median	32,25	36,97	39,73	38,76	35,55	30,63	51,89	71,26	75,35	76,20	57,90

75% Percentile	40,20	51,07	54,70	57,45	49,30	54,72	57,87	92,29	82,52	82,61	65,08
Maximum	48,31	113,6	81,15	108,8	85,81	55,69	63,29	118,4	91,87	90,79	67,02
Range	21,08	84,43	56,74	93,75	62,16	30,12	27,99	54,07	34,47	27,78	30,38
Mean	34,61	47,92	44,61	45,32	41,90	37,42	50,41	79,00	72,84	75,98	54,24
Std. Deviation	7,559	27,80	18,56	28,00	22,05	13,79	9,831	20,71	13,32	9,406	12,70
Std. Error of Mean	3,086	9,827	7,015	9,333	9,004	5,632	4,014	8,453	5,439	3,840	5,184
